# Arid4b physically interacts with Tfap2c in mouse embryonic stem cells

**DOI:** 10.3906/biy-2010-67

**Published:** 2021-04-20

**Authors:** Ezgi Gül KESKİN, Jialiang HUANG, Nihal TERZİ ÇİZMECİOĞLU

**Affiliations:** 1 Department of Biological Sciences, Faculty of Arts and Sciences, Middle East Technical University, Ankara Turkey; 2 State Key Laboratory of Cellular Stress Biology, Innovation Center for Cell Signaling Network, School of Life Sciences, Xiamen University, Xiamen, Fujian China

**Keywords:** Chromatin, epigenetics, embryonic stem cell, lineage commitment, transcription factor

## Abstract

Precise regulation of gene expression is required for embryonic stem cell (ESC) differentiation. Transcription factor (TF) networks coordinate the balance of pluripotency and differentiation in response to extracellular and intracellular signals. Chromatin factors work alongside TFs to achieve timely regulation of gene expression for differentiation process. Our previous studies showed that a member of the Sin3a corepressor complex, Arid4b, is critical for proper mouse ESC differentiation into mesoderm and endoderm. We found elevated histone 3 lysine 27 acetylation (H3K27Ac) in a subset of genomic loci in meso/endoderm directed arid4bΔ cells, coincident with their derepression. We reasoned that Sin3a complex may be required for the suppression of these genes during differentiation. To identify TFs that might cooperate with Arid4b for this function, we found consensus TF binding sequences enriched in H3K27Ac elevated regions in arid4bΔ cells. Of these candidate TFs, we validated expression of Bach1, Ddit3, Prrx2, Znf354c and Tfap2c in mESCs. We then demonstrated a physical interaction between Arid4b and Tfap2c in mESCs using endogenous coimmunoprecipitation and proximity ligation assay experiments. Our results point to a role of Arid4b in the Sin3a complex in repression of a subset of Tfap2c-regulated genes during meso/endoderm differentiation.

## 1. Introduction

Embryonic stem cells (ESC) are derived from inner cell mass (ICM)s (Takaoka and Hamada, 2012). ESCs have self-renewal ability and pluripotency, which makes them very valuable for studying molecular mechanisms of cell differentiation in vitro (Gaspar-Maia, et al., 2011). Core pluripotency transcription factors (Oct4, Nanog Sox2, Klf4, c-Myc and others) maintain the balance of pluripotency in ESCs in coordination with chromatin factors (Orkin and Hochedlinger, 2011).

Eukaryotic DNA is wrapped around nucleosomes that contain conserved histone proteins. Chromatin environment is important for the execution of gene expression programs upon response to differentiation stimuli. Through cooperation between lineage specific TFs and chromatin factors, hundreds of differentiation related genes are activated whereas the ESC specific gene expression program is suppressed in a timely and synchronized manner (Orkin and Hochedlinger, 2011).

Sin3a is a corepressor complex that is important for early development and efficient self-renewal and somatic cell reprogramming (Liang et al., 2008). Sin3a complex includes histone deacetylases Hdac1 and Hdac2 (Kelly and Cowley, 2013). It was shown that Sin3a/Hdac1 complex and core pluripotency protein Nanog physically interacts with each other in ESCs (Liang et al*.*, 2008). Arid4b is a nonenzymatic member of the Sin3a complex. Its ARID domain provides weak DNA interaction whereas the chromodomain and tudor domains play a role in interaction with modified histones. Arid4b might be responsible for the recruitment of the Sin3a complex to DNA and the stability of the complex through sequence nonspecific DNA or histone interactions (Fleischer et al., 2003).

We have previously identified Arid4b as a critical factor for mouse ESC differentiation towards meso/endoderm (Terzi Cizmecioglu et al*.*, 2020). Arid4b loss did not alter the mESC proliferation or cell death but resulted in decreased cell proliferation and elevated cleaved caspase-3 upon onset of endoderm differentiation (Guven and Terzi Cizmecioglu, 2021). Gene set enrichment analyses (GSEA) of arid4b∆ vs. WT endoderm (or mesoderm) differentiated cells did not show an enrichment of apoptosis, senescence or autophagy pathways but rather showed loss of cell fate specification and embryonic patterning pathways in arid4b∆ cells, indicating that the changes in cell death mechanisms might not be a major contributor towards Arid4b function during mESC differentiation. We, therefore, focused on the mechanism of how Arid4b executes cell fate related gene expression changes. Arid4b-deficient mESCs showed altered H3K27me3 and H3K27ac in lineage specific genes (Terzi Cizmecioglu et al*.*, 2020). Regulation of gene expression by transcription factors (TFs) depend on the target gene chromatin environment. We reasoned Arid4b in the Sin3a complex may prepare a suitable chromatin environment for TFs to properly execute meso/endodermal gene expression program. Alternatively, the Sin3a complex might be recruited by TFs to target lineage specific genes for mESC differentiation. Therefore, we set out to identify TFs that might functionally and physically interact with Arid4b in mESCs. 

## 2. Materials and methods 

### 2.1. Prediction of consensus TF binding sequences 

To identify enriched motifs within a subset of genomic loci, we performed motif enrichment analysis using HOMER (Heinz et al*.*, 2010), as previously described in Cai et al*.* (2020). The position weight matrixes (PWM) of core vertebrate motifs were downloaded from the JASPAR databaseJASPAR (2020). JASPAR Database [online]. Website http://jaspar.genereg.net/ [accessed 10 January 2017]. . The enrichment score of each motif in a subset of genomic loci was defined as –log10(p-value), where p-value corresponds to the significance of observed overrepresentation of each motif site in the subset of target genomic loci compared to control regions randomly selected. The significantly enriched motifs with an enrichment score of at least 10 were selected. 

### 2.2. Cell culture

WT (CJ9) and arid4bΔ mESCs were expanded as in Terzi Cizmecioglu et al*.* (2020). These cells were used qRT-PCR experiments to determine the expression level of candidate TFs. Due to difficulties in the CF-1 MEF import, we have established and optimized MEF-free culture of mESC culture, similar toYing et al*.* (2008). We optimized the ESC maintenance in 2i+LIF containing defined medium suggested by the protocol in Mulas et al*.* (2019). We confirmed that WT mESCs grown in 2i+LIF conditions can be differentiated to endoderm in similar kinetics as the traditional LIF containing serum medium conditions. We also validated that mESCs in 2i+LIF medium are pluripotent and does not demonstrate expression of neuroectoderm marker Sox1 (not shown). The defined media contains 50% Neurobasal (Gibco, Thermo Fisher Scientific, Waltham, MA, USA), 50% DMEM-F12 (Gibco, Thermo Fisher Scientific), 0.5% N-2 supplement (100X) (Thermo Fisher Scientific), 1% 50× B-27 supplement (Thermo Fisher Scientific), 0.5% BSA (Sigma-Aldrich, St. Louis, MO, USA), 1% GlutaMAX supplement (Gibco, Thermo Fisher Scientific), 1% penicillin-streptomycin (10.000 U/mL) (Thermo Fisher Scientific), monothioglycerol (1.5 × 10–4 M final) (Sigma-Aldrich) and 4% FBS (Thermo Fisher Scientific). The media was supplemented with 3 µM CHIR99021 (Selleck Chemicals, Houston, TX, USA), 1 µM PD0325901 (Selleck Chemicals) and 1% LIF (Millipore, Burlington, MA, USA). When cells were 70%–80% confluent, cells were split by using TrypLE Express Enzyme (1×) (Thermo Fisher Scientific). Whole cell extracts for Western blot and nuclear extracts for coimmunoprecipitation were prepared from these cells.

During ESC and endoderm differentiation protocols, cell growth and viability was routinely assessed by trypan blue staining, followed with counting using Countess II Automated Cell Counter (ThermoFisher Scientific). 

### 2.3. qPCR analysis

Cells were collected and resuspended in TRIzol (15596018; ThermoFisher Scientific). RNA was extracted using Qiagen RNeasy plus kits (Qiagen, Valencia, CA, USA) according to provided protocols. Concentration of the purified RNA samples were tested on Nanodrop. Equal amount of total RNA (250 ng–1µg) was converted into cDNA using iScript cDNA synthesis kit (1708890; Bio-Rad Laboratories, Hercules, CA, USA). The qPCR primers of candidate TFs were obtained from previously used studies. The primer sequence and PubMed ID of the articles which includes primer pairs are shown in the Table 1. qPCR was performed with SsoAdvanced Universal SYBR Green Supermix (Bio-Rad Laboratories) using BioRad CFX Connect machine according to manufacturer’s protocols. Melting curve for all primers that were used in qRT-PCR was performed to check the specificity of the primer pairs. The expression level of candidate TFs was represented as percentage of b-actin. 

**Table 1:** Primers used in the study, along with the PubMed ID (PMID) of the original paper they were obtained from.

Primer name	Sequence (5’-> 3’)	PMID
b-actin-F-qPCR	ATGAAGATCCTGACCGAGCG	14998924
b-actin-R-qPCR	TACTTGCGCTCAGGAGGAGC	14998924
Pdx1-F-qPCR	CAACATCACTGCCAGCTCCACC	27715254
Pdx1-R-qPCR	TCACCTCCACCACCACCTTCCA	27715254
Prrx2-F-qPCR	AGTGAGGCACGTGTCCAAGTC	24770895
Prrx2-R-qPCR	GTAGCCAGCATGGCACGTT	24770895
Bach1-F-qPCR	TGAGTGAGAGTGCGGTATTTGC	26084661
Bach1-R-qPCR	GTCAGTCTGGCCTACGATTCT	26084661
MafK-F-qPCR	GACAGGGCCCGGGTTATG	10409670
MafK-R-qPCR	AGCTCATCATCGCTAAGAACAGG	10409670
Znf354c-F-qPCR	CCGGCGTCCGCATATTT	27723809
Znf354c-R-qPCR	CCCTTCTTAGTTTTTCTGCCAAAG	27723809
Mzf1-F-qPCR	GAGGCTGCTGCCCTAGTAGA	22246292
Mzf1-R-qPCR	GAGGGCTCCATCTTCTCTGA	22246292
Ddit3-F-qPCR	GTCAGTTATCTTGAGCCTAACACG	26634309
Ddit3-R-qPCR	TGTGGTGGTGTATGAAGATGC	26634309
Tfap2c-F-qCPR	GGGCTTTTCTCTCTTGGCTGGT	23913270
Tfap2c-R-qCPR	TCCACACGTCACCCACACAA	23913270
Atoh1-F-qPCR	ATGCACGGGCTGAACCA	26786414
Atoh1-R-qPCR	TCGTTGTTGAAGGACGGGATA	26786414
Stat6-F-qPCR	GCATCTATCAGAGGGACCCC	26899911
Stat6-R-qPCR	ACTTGTCCAGTCTTAGGCCC	26899911

### 2.4. Western blot

1 × 106 wild-type and arid4b∆ grown and lysed directly in 2x Laemmli buffer (Bio-Rad Laboratories) including β-mercaptoethanol at 95 °C for 10 min. After centrifugation to get rid of the cell debris, equal amounts of cell lysate were loaded on 12% polyacrylamide gel. Bio-Rad Clarity Western ECL was used with Bio-Rad ChemiDoc MP Imaging System. Bio-Rad Clarity Max Western ECL which is an amplified form of ECL was used for antibodies that has weak signal. The antibodies used in the study are listed in Table 2. The quantitation of the Western blot experiments were done using ImageJ. The numerical values were graphed using GraphPad Prism. Unpaired t-test was used for statistical analysis.

**Table 2:** Antibodies used in the study.

Protein name	Brand/cat. no.	Host	Dilution
Bach1	Abcam/ ab124919	rabbit	1:1000
Znf354c	LifeSpan/LS-C145172-100	rabbit	1:500
Tfap2c	Sigma/sab2102408	rabbit	1:1000
Tfap2c	Santa Cruz/ sc-12762	mouse	1:100
Ddit3	Santa Cruz/sc-7351	mouse	1:1000
Prrx2	Abcam/ab156096	rabbit	1:100
Actin	Millipore/mab1501	mouse	1:3000
Arid4b	Bethyl/A302-233A	rabbit	1:5000
Sin3a	Active Motif/ 39866	rabbit	1:5000
Anti-rabbit IgG H&L (HRP)	Abcam/ab97051	goat	1:5000
Anti-mouse IgG H&L (HRP)	Abcam/ab97023	goat	1:5000

### 2.5. Coimmunoprecipitation

Nuclear extracts were prepared from wild-type (CJ9) and arid4b∆ mESCs using the Universal Magnetic Co-IP Kit (catalog no: 54002; Active Motif, Carlsbad, CA, USA) according to manufacturer’s protocol. For coimmunoprecipitation, kit protocol was followed with the following optimizations: 400 µg of nuclear extract was incubated with 5 µg anti-Arid4b (A302-233A; Bethyl Laboratories, Montgomery, TX, USA) antibody. Nuclear extracts were precleared on protein-G coated magnetic beads for 2 h at +4 °C on the rotator. After immunoprecipitation, three extra washes in NP-40 buffer was used to decrease nonspecific background signal. Twenty micrograms nuclear extract from each sample was taken as input. After immunoprecipitation and washes, beads were boiled in 2x Laemmli Buffer (Bio-Rad Laboratories) supplemented with β-mercaptoethanol at 95 °C for 10 min. Normal rabbit IgG was used as a negative control. As another control, Arid4b protein was precipitated in the nuclear extract obtained from arid4bΔ mESCs. 

The heavy chain of IgG (50 kD) overlaps with Tfap2c. Therefore, the membrane that included Tfap2c was incubated with conformational specific secondary antibody (CST, L27A9) for 1 h at RT. Then, it was incubated with tertiary antibody for 1 h at RT because the conformational specific antibody was not conjugated to HRP. Bio-Rad Clarity Western ECL was used with Bio-Rad ChemiDoc MP Imaging System for visualization. Bio-Rad Clarity Max Western ECL was used for antibodies that has weak signal. The quantitation of the coimmunoprecipitation results were done using ImageJ. The numerical values were graphed using GraphPad Prism.

### 2.6. Proximity ligation assay

PLA is performed using Duolink In Situ Red Starter Kit (Sigma-Aldrich, DUO92101) using Arid4b (rabbit, 1:500, ls-c199776; LifeSpan BioSciences, Seattle, WA, USA) and Tfap2c (mouse, 1:100, sc12762; Santa Cruz Biotechnology, Dallas, TX, USA) primary antibodies on wild-type (CJ9) mESCs according to the manufacturer’s protocol. Confocal microscopy (Leica TCS SP8, Leica Microsystems, Wetzlar, Germany) imaging was performed at Bilkent University UNAM Laboratories (Ankara, Turkey).

## 3. Results

### 3.1. Prediction of gene expression profile of candidate TFs 

**Figure 1:**
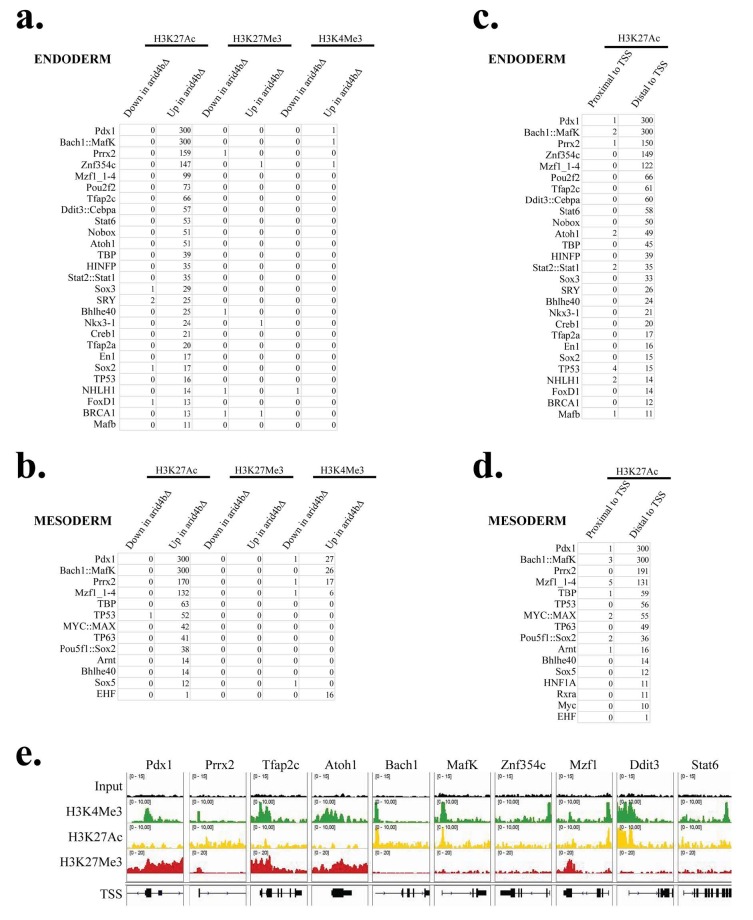
Transcription factor motif enrichment in differential chromatin regions of differentiated WT and arid4bΔ cells. Transcription factor motif enrichment analysis of a. endoderm directed and b. mesoderm directed wild-type (CJ9) and arid4bΔ cells. Regions that have higher or lower level of each modification (H3K27Ac, H3K27Me3 and H3K4Me3) were previously determined using ChIP. Regions that have elevated H3K27Ac level in arid4bΔ cells were analyzed for transcription motif enrichment based on distance to the transcription start site (TSS) (proximal vs. distal) in c. endoderm or d. mesoderm differentiation. e. ChIP profile of H3K4Me3, H3K27Ac and H3K27Me3 across candidate TFs in WT (CJ9) cells.

We have previously shown that Arid4b loss results in altered chromatin modifications coincident with a meso/endodermal differentiation defect (Terzi Cizmecioglu et al., 2020). We reasoned that the function of Arid4b is coordinated with functional, and possibly physical, interactions with TFs during mESC differentiation. Therefore, we sought out consensus TF binding sequences that are enriched in regions with altered H3K4me3, H3K27me3 or H3K27Ac modifications in endoderm or mesoderm directed arid4b∆ cells (Figures 1a and 1b). For both lineages, we identified TF binding sequence enrichment primarily in regions with elevated H3K27Ac in arid4b∆ cells whereas no significant enrichment was observed for other modifications. Elevated H3K27Ac correlates with aberrant transcriptional activation in meso/endoderm directed arid4b∆ cells, suggesting that the role of Arid4b in mESC lineage commitment might be at least partially explained by the suppression of these genes (Terzi Cizmecioglu et al*.*, 2020). This is also consistent with the molecular role of Arid4b as part of a histone deacetylase complex. Therefore, we wanted to figure out whether Arid4b shares a physical interaction with any of these candidate TFs. 

H3K27Ac is found in the enhancer and promoters of active genes. Super-enhancers with high level of H3K27Ac are important in the expression of genes involved in cell identity (Denes et al*.*, 2013; Whyte et al*.*, 2013; Hnisz et al*.*, 2015). Consistently, we identified that the enriched TF binding sequences fall into distal regulatory regions rather than proximal regions (Figures 1c and 1d). 

Of these ~30 TF binding sequences, some TFs might not be expressed in mESCs or might not have a function in cell fate determination. We reviewed each candidate TF based on its expression during mouse embryonic development or ESCs (BioGPSThe Scripps Research Institute (2021). BioGPS Website and Database [online]. Website http://biogps.org [accesed 10 January 2018]. and LifeMapLifeMap Sciences, Inc. (2021). LifeMap Discovery [online]. Website https://discovery.lifemapsc.com [accessed 10 January 2018].), on its known functional or physical interaction with the Sin3a complex members, or on its known involvement with cell fate decisions. 

After this elimination, we predicted the expression of candidate TFs based on H3K4Me3, H3K27Me3 and H3K27Ac level along their gene loci. Using our ChIP-seq data in Integrative Genomics Viewer (IGV)Broad Instituteand the Regents of theUniversity of California (2018). Integrative Genomics Viewer [online]. Website https://software.broadinstitute.org/software/igv/ [accessed 10 February 2018]., we visualized these modifications (Figure 1e). Gene loci that are marked with H3K4Me3 and/or H3K27Ac but with little/no H3K27Me3 such as Prrx2, Bach1, MafK, Znf354c, Ddit3 and Stat6 were predicted to be transcriptionally active. On the other hand, Pdx1, Tfap2c, Atoh1 and Mzf1 had H3K27Me3 along with H3K4Me3, suggestive of bivalent chromatin structure and low level expression.

### 3.2. Expression of candidate TFs in wild-type and arid4bΔ mESCs 

**Figure 2:**
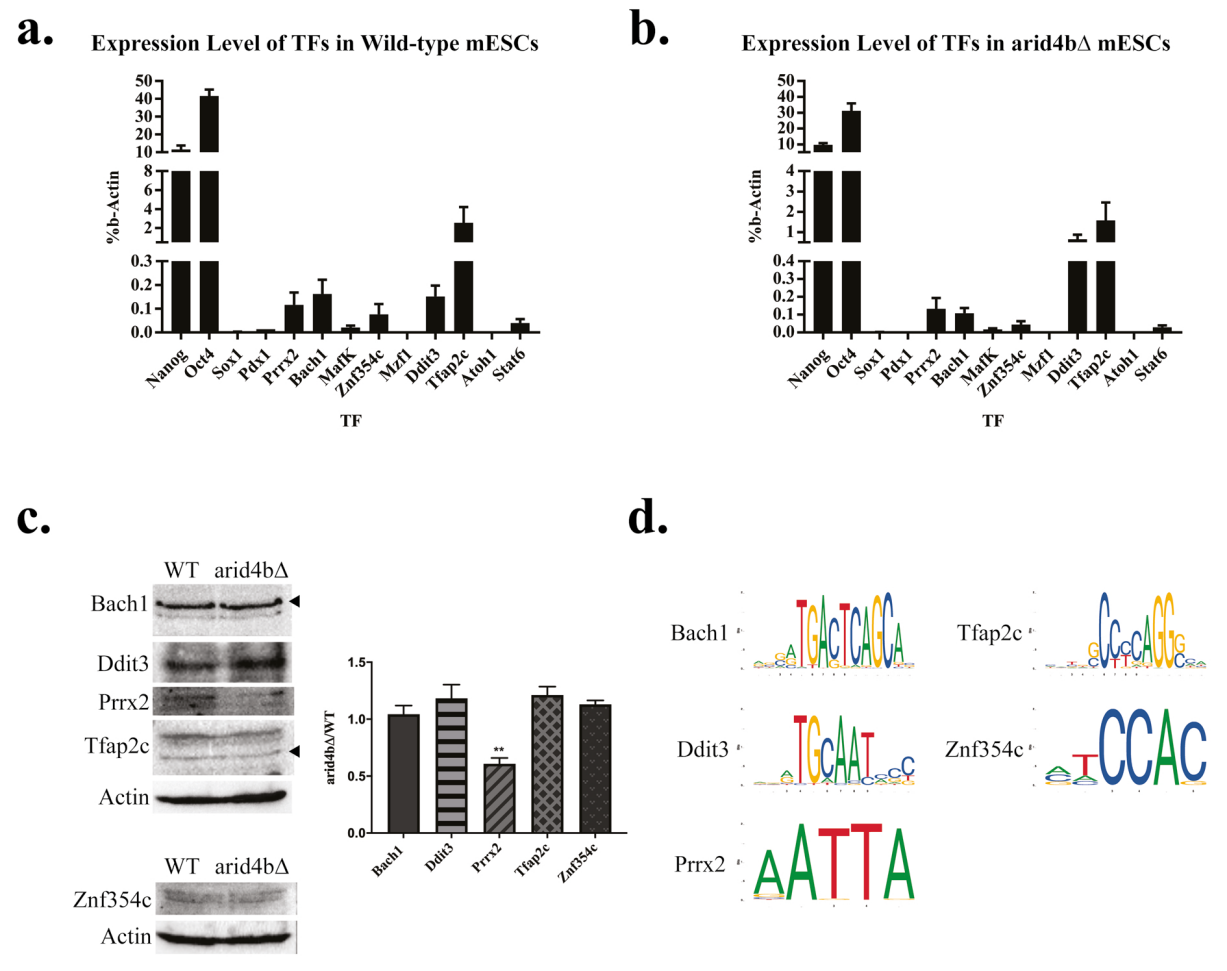
Expression of candidate transcription factors in WT and arid4bΔ mESCs. Expression of ten candidate transcription factors (TFs) (Pdx1, Prrx2, Bach1, MafK, Znf354c, Mzf1, Ddit3, Tfap2c, Atoh1 and Stat6) were determined by RT-qPCR in a. wild-type (CJ9) b. arid4bΔ mESCs. ESC-specific TFs Nanog and Oct4 were used as positive controls whereas neuroectoderm-specific TF Sox1 was used as a negative control for comparison. Error bars represent standard error of three independent biological replicates. c. Expression of Bach1, Ddit3, Prrx2, Tfap2c and Znf354c were determined using Western blot analysis in WT and arid4bΔ mESCs. Actin was used as a loading control. The results are representative of three independent biological replicates. The quantification of Western blot replicates was performed using ImageJ. The values were first normalized to Actin and then represented as arid4bΔ over wild-type (WT). Error bars represent standard error. All values except Prrx2 (p-value < 0.005) were found nonsignificant in arid4bΔ vs. wild-type by unpaired t-test. d. Consensus DNA binding sequence representations of Bach1, Ddit3, Prrx2, Tfap2c and Znf354c (JASPAR).

We wanted to experimentally validate our predictions from Figure 1e. We performed RT-qPCR analyses for candidate TFs in wild-type mESCs and arid4b∆ mESCs (Figures 2a and 2b). Nanog and Oct4 pluripotency TFs were used as positive controls whereas neuro-ectodermal TF Sox1 was used as a negative control. Prrx2, Bach1, Znf354c, Ddit3 and Tfap2c expression was observed at varying levels in mESCs. Pdx1, MafK, Stat6 were expressed at very low levels whereas Atoh1 or Mzf1 were not expressed. Pdx1, MafK, Stat6, Atoh1 and Mzf1 were eliminated from further interaction studies. Interestingly, the predicted low expression of Tfap2c based on its bivalent state did not correlate with its actual transcript abundance (Figure 1e vs. Figure 2a). We posit additional histone acetylations on H3 or H4 might contribute to higher transcriptional efficiency of Tfap2c or that its transcript has elevated stability.

We also verified the expression of Bach1, Tfap2c, Ddit3, Prrx2, Znf354c TFs on protein level in wild-type and arid4bΔ mESCs using Western blot (Figure 2c). Multiple sized bands were observed for Bach1 and Tfap2c. All tested proteins except Prrx2 were found to be similarly expressed in wild-type and arid4b∆ mESCs, suggesting Arid4b might affect Prrx2 expression. Although we were able to verify the expression of Znf354c, its expression in mESCs was very low. Due to anticipated difficulties for immunoprecipitation experiments, we eliminated Znf354c from further analysis.

**Figure 3:**
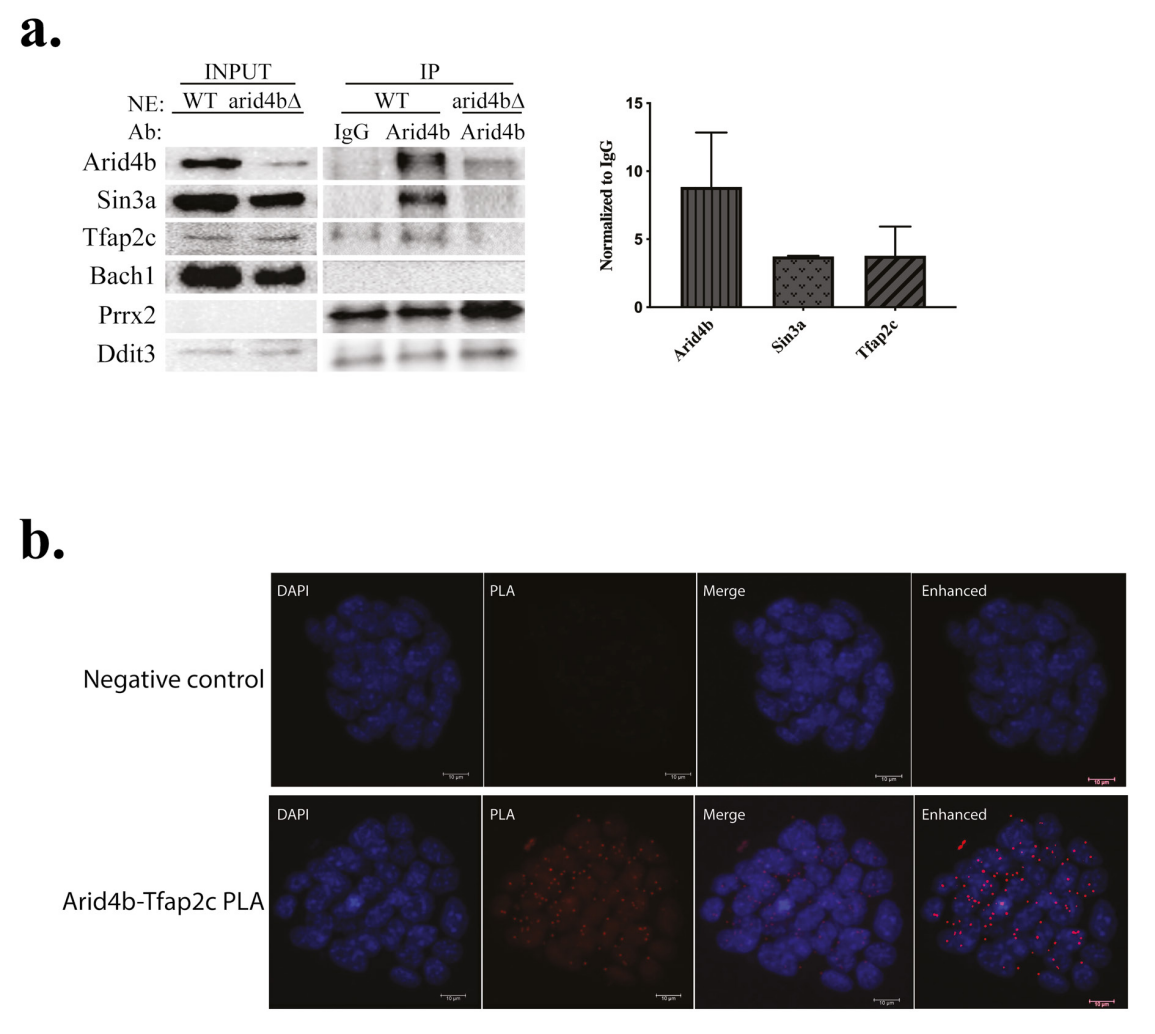
Physical interaction between Arid4b and candidate TFs. a. Coimmunoprecipitation experiments were performed in WT (CJ9) or arid4bΔ mESC nuclear extracts (NE) using Arid4b antibody (and rabbit IgG as a control). Bands for Arid4b, Sin3a and Tfap2c were quantified using ImageJ. The IP values were normalized to input and represented as a ratio of antibody IP over IgG IP. The results are representative of at least three independent biological replicates. b. Proximity ligation assay (PLA) of Arid4b (rabbit) and Tfap2c (mouse) (bottom panels) in WT (CJ9) mESCs. Red dots (Texas Red) depict intracellular interactions between Arid4b and Tfap2c. Experimental negative control (top panels) is PLA reaction without primary antibodies.

Based on our expression data in mESCs, Bach1, Tfap2c, Ddit3 and Prrx2 whose consensus binding DNA sequences shown in Figure 2d, were chosen to be tested for physical interaction with the Sin3a complex member Arid4b. The bands corresponding to each TF was confirmed according to size as well as the positions in our later experiments using nuclear extracts (Figure 3a) and labeled with arrowheads in the figure.

### 3.3. Determination of physical interaction between Arid4b and candidate TFs

We first wanted to analyze a possible physical interaction between Arid4b and selected TFs using endogenous coimmunoprecipitation (co-IP) experiments. In an effort to minimize nonspecific protein-protein interactions, we performed co-IPs from mESC nuclear extracts, where the Sin3a complex is functionally active. We performed IgG IP from wild-type and Arid4b antibody IP from arid4b∆ mESC nuclear extracts as negative controls. Even though a weak band for Arid4b was observed in Arid4b antibody IP from arid4b∆ mESC nuclear extract, it does not seem to interact with Sin3a, confirming it does not correspond to Arid4b and is a nonspecific pull-down (Figure 3a). 

As expected, Arid4b successfully coimmunoprecipitated Sin3a. Slightly more Tfap2c was coimmunoprecipitated with Arid4b than with IgG. The specificity of the co-IP between Arid4b and Tfap2c was further supported by the absence of Tfap2c pull-down from Arid4b IP using arid4b∆ mESC nuclear extracts. These results suggest specific, albeit weak or transient interaction between Arid4b and Tfap2c in mESCs. 

No specific interaction was observed between Arid4b and Bach1 or Ddit3, even though both proteins were found in the input nuclear extracts. Prrx2 protein was detected in whole cell lysate of wild-type and arid4bΔ mESCs by Western blot experiments. However, it was not easily detected in nuclear extract input (Figure 3a), suggesting primarily cytoplasmic localization of Prrx2. Since similar amounts of Prrx2 protein was coimmunoprecipitated in both experimental and negative controls, we conclude no specific interaction between Arid4b and Prrx2. 

To validate the physical interaction between Arid4b and Tfap2c, we performed proximity ligation assay (PLA) in wild-type mESCs (Figure 3b). PLA is a highly sensitive technique in detection of protein-protein interactions in situ. The use of two complementary probe linked primary antibodies will only produce signal (red dots in the figure) when the distance between the antibodies (and thus the target proteins) is closer than 40 nm. Consistent with co-IP experiments, we demonstrated a physical interaction with Arid4b and Tfap2c. The majority of the signal coincided with DAPI signal, indicating that the interaction occurs within the nuclei where the Sin3a complex is functionally active. 

Taken together, our results show a physical interaction between Arid4b and Tfap2c and point to a role of Arid4b in the suppression of at least a subset of Tfap2c regulated genes during mESC lineage commitment. 

## 4. Discussion 

Pluripotency state of mESC is maintained by the key transcription factors such as Oct4, Nanog and Sox2. TFs works with chromatin factors to maintain pluripotency (Orkin and Hochedlinger, 2011). We previously identified chromatin factors that have a critical role in differentiation of mESCs towards mesoderm and endoderm (Terzi Cizmecioglu et al*.*, 2020). One such factor is the chromatin reader Arid4b which is a member of the Sin3a corepressor. In this study, we aimed at identifying any possible TF partners working alongside Arid4b for its function in mESC differentiation. We hypothesized that Arid4b may work with TFs to regulate differentiation process. Therefore, we started out with the identification of consensus TF binding sites in regions with altered chromatin modifications in arid4bΔ cells. It was previously shown that Sin3a corepressor complex is targeted to enhancer regions for transcription repression (Carleton et al., 2017). We also reported an increase in the number of super enhancers in meso/endoderm directed arid4b∆ cells (Terzi Cizmecioglu et al*.*, 2020). Consistent with these finding, we identified enrichment of numerous TF binding sequences specifically in transcription start site distal regions with elevated H3K27Ac in arid4b∆ cells directed towards meso/endoderm. Since high H3K27Ac over super-enhancer regions are important for stability of cell identity and determination of cell fate (Denes et al*.*, 2013; Whyte et al*.*, 2013; Hnisz et al*.*, 2015). We reasoned the identified TFs might be central to Arid4b control over mESC lineage commitment. The majority of these identified TF binding sequences were common among mesoderm and endoderm differentiation, consistent with the common origins of these embryonic lineages. Of the various TFs, we confirmed the expression of seven (Prrx2, Tfap2c, Bach1, MafK, Stat6, Znf354c and Ddit3) both in mRNA and protein level in WT and arid4b∆ mESCs. Out of these, only Prrx2 expression was altered in arid4b∆ mESCs, suggesting Arid4b might be involved in Prrx2 regulation.

The overrepresentation of a TF binding sequence or the expression of that particular TF in mESCs does not necessarily implicate its physical interaction with Arid4b. It is conceivable that these TFs might affect mESC state or differentiation through other mechanisms without any physical interaction with Arid4b. The lack of physical interaction between Arid4b and Prrx2, Ddit3 or Bach1 suggests such mechanisms and needs to be further studied. 

Using co-IP and PLA experiments, we demonstrated a novel physical interaction between Arid4b and Tfap2c. It was previously shown that Tfap2c is involved in cell fate determination, specifically towards primordial germ cell (PGC) lineage through cooperation with Blimp1 and Prdm14 (Magnúsdóttir et al*.*, 2013). Additionally, it is suggested that Tfap2c acts downstream of Nanog in PGC specification in vitro (Murakami et al., 2016). Another study on human embryonic stem cells (hESC) showed that Tfap2c maintains pluripotency by enabling access to OCT4 enhancer region (Pastor et al*.*, 2018). These findings support Tfap2c as an important factor in cell fate determination. It will be interesting to figure out a possible role of Tfap2c in meso/endoderm commitment andunderstand how the Sin3a complex mechanistically works with Tfap2c in mESCs. 
